# A Comprehensive Review on Bioactive Molecules and Advanced Microorganism Management Technologies

**DOI:** 10.3390/cimb46110789

**Published:** 2024-11-20

**Authors:** Adil Farooq Wali, Sirajunisa Talath, Sathvik B. Sridhar, Javedh Shareef, Manjunatha Goud, Imran Rashid Rangraze, Nowar Nizar Alaani, Omnia Ibrahim Mohamed

**Affiliations:** 1 Department of Pharmaceutical Chemistry, RAK College of Pharmacy, RAK Medical and Health Sciences University, Ras Al Khaimah 11172, United Arab Emirates; 2 Department of Clinical Pharmacy and Pharmacology, RAK College of Pharmacy, RAK Medical and Health Sciences University, Ras Al Khaimah 11172, United Arab Emirates; sathvik@rakmhsu.ac.ae (S.B.S.); javedh@rakmhsu.ac.ae (J.S.); 3 Department of Biochemistry, RAK College of Medical Sciences, RAK Medical and Health Sciences University, Ras Al Khaimah 11172, United Arab Emirates; manjunatha@rakmhsu.ac.ae; 4 Department of Internal Medicine, RAK College of Medical Sciences, RAK Medical and Health Sciences University, Ras Al Khaimah 11172, United Arab Emirates; imranrashid@rakmhsu.ac.ae; 5 Department of General Education, RAK Medical and Health Sciences University, Ras Al Khaimah 11172, United Arab Emirates; nowar@rakmhsu.ac.ae (N.N.A.); omnia@rakmhsu.ac.ae (O.I.M.)

**Keywords:** drug discovery, microorganism, natural products, phytoconstituents, antimicrobial resistance

## Abstract

The advent of new strains of resistant microbes and the concomitant growth in multidrug resistance have made antimicrobial resistance an urgent public health concern. New antimicrobials are desperately needed to boost the success rates of treating infectious diseases and save lives. There are many intriguing biomolecules with antibacterial action, which are mostly unexplored in microorganisms. This review article describes the importance of natural compounds against microorganisms using advanced techniques to protect individuals from diseases. We have conducted an extensive literature review using databases such as SCOPUS, SCI, PUBMED, ScienceDirect, and Medline to gather relevant information. Our review covers various microorganism sources for antimicrobials, antifungal drugs, micro-culturing techniques, and microbial-based microsystems’ applications. Every kind of higher trophic life depends on microorganisms for sustenance. The unseen majority is essential to understanding how humans and other living forms can survive anthropogenic climate change. The article discusses antimicrobial substances and the latest techniques and strategies for developing effective treatments. Novel model systems and cutting-edge biomolecular and computational methodologies could help researchers enhance antimicrobial resistance by completely capitalizing on lead antimicrobials.

## 1. Introduction

Multidrug-resistant organisms and antimicrobial-resistant diseases present a growing threat to public health and the healthcare system, as they undermine the effectiveness of standard treatments. Each year, thousands of individuals die from infections caused by resistant pathogens, as these diseases are becoming harder to treat with existing medications [1]. New strains of resistant microbes refer to microbial species, particularly bacteria, that have developed resistance to multiple classes of antimicrobial drugs, rendering them difficult or impossible to treat with standard therapies. These strains have evolved through genetic mutations, horizontal gene transfer, or selective pressure resulting from the overuse or misuse of antibiotics. Multidrug-resistant (MDR) microbes include Methicillin-resistant *Staphylococcus aureus* (MRSA), Carbapenem-resistant *Enterobacteriaceae* (CRE), Vancomycin-resistant *Enterococci* (VRE), Multidrug-resistant *Mycobacterium tuberculosis* (MDR-TB), and Extended-spectrum beta-lactamase (ESBL)-producing bacteria. *Staphylococcus aureus* is a bacterium commonly found on the skin and within the nasal passages of healthy people. In most cases, it is harmless but can also cause minor skin infections and even more serious diseases like pneumonia and bloodstream infections. Conversely, MRSA is a specific species of *Staphylococcus aureus* that has evolved resistance to methicillin and other beta-lactam antibiotics. MRSA infections, therefore, prove more challenging to treat; indeed, this resistance often means the infections are more severe, particularly within healthcare settings or among immunocompromised patients. In addition, MRSA falls into two subcategories where one is hospital-acquired, specifically associated with hospital and healthcare settings that generally targets old patients or patients suffering from some illness, and the other type is defined as community-associated MRSA that primarily infects young patients with healthy immunity but without specific risk factors to target the section of the population. Generally, treatment of MRSA involves alternative antibiotics in the form of vancomycin or linezolid, whereas the treatment with usual antibiotics is performed in the case of non-resistant *Staphylococcus aureus.* So, though MRSA is essentially *Staphylococcus aureus*, the differentiation between these two has significantly varied their treatment and the profile of associated risk factors. These strains’ rapid emergence and spread and the lack of new antibiotics have made antimicrobial resistance (AMR) an urgent public health crisis. Without effective interventions, infections from these resistant microbes could lead to increased mortality, longer hospital stays, and higher healthcare costs [2]. Consequently, it is crucial to investigate potential replacements for current antimicrobials. They account for over two-thirds of all newly authorized pharmaceutical applications [3].

In contrast to antibiotics produced by microorganisms, plant-based antimicrobials have attracted many researchers due to their promising effects with fewer side effects. Bioactive chemicals with antibacterial, antifungal, and cytotoxic bioactivity are produced by microorganisms [4,5,6,7]. Once again, their ability to produce functionally rich secondary metabolites contributes to their success in various habitats. Due to their unique biological features, microorganisms have recently attracted researchers’ interest as a potential source of new antimicrobial drugs [8]. Modern molecular biology, genetic, genomic, and computational approaches make mining microbial structure systems for drug discovery easier [9,10,11].

Viruses, bacteria, archaea, fungi, and protists are all examples of microorganisms, which are biotic, ubiquitous, and diverse entities. Novel antimicrobial agents are primarily discovered in bacteria and fungi. Clinically resistant germs like *Staphylococcus aureus*, *Methicillin-resistant Staphylococcus aureus*, *Micrococcus luteus*, *Bacillus subtilis*, *E. faecalis*, and *Enterococcus faecalis* are toxic to cyclic peptides, polyketides, and alkaloids found in bacteria, such as *mathiapeptide A*, *destotamide B*, *Marfomycins A*, *B*, and *E*, *spirotetronates polyketides*, *abyssomycin C*, *Lobophorin* F and H, and sesquiterpene derivatives, alkaloids, and *mafuraquinocins A and D*, which are derived from bacterial sources. *Streptomyces scopuliridis* produces the cyclic peptide Desotamide B, effective against Staphylococcus aureus [12]. *Marinactinospora thermotolerans* generates Marthiapeptide A, which shows antimicrobial activity against *S. aureus*, *Micrococcus luteus*, *Bacillus subtilis*, and *Bacillus thuringiensis*. Verrucosispora spp. produces the spirotetronate polyketide Abyssomicin C, targeting methicillin- and vancomycin-resistant *S. aureus. Streptomyces drozdowiczii* creates Marfomycins A, B, and E, effective against *M. luteus* [13]. Streptomyces spp. synthesizes Lobophorin H and F, spirotetronate polyketides active against *B. subtilis*, *S. aureus*, and *Enterococcus faecalis* [14]. Additionally, *Streptomyces niveus* produces the sesquiterpene derivatives Marfuraquinocin A and D, targeting methicillin-resistant *S. aureus*, while Streptomyces spp. produces the alkaloid Caboxamycin, which inhibits *S. epidermis*, *S. lentus*, and *B. subtilis* [15].

Inhibitors of Gram-negative and Gram-positive pathogenic bacteria include fungus-derived xanthones, emerixanthones, and engyodontiumsones. Analogs of ambuic acid, such as penicyclone and depsidone, have this property [16]. Yeast, microalgae, and cyanobacteria are among the other groups whose compounds have demonstrated anti-infective potential in animal and laboratory studies [17].

**Figure 1:**
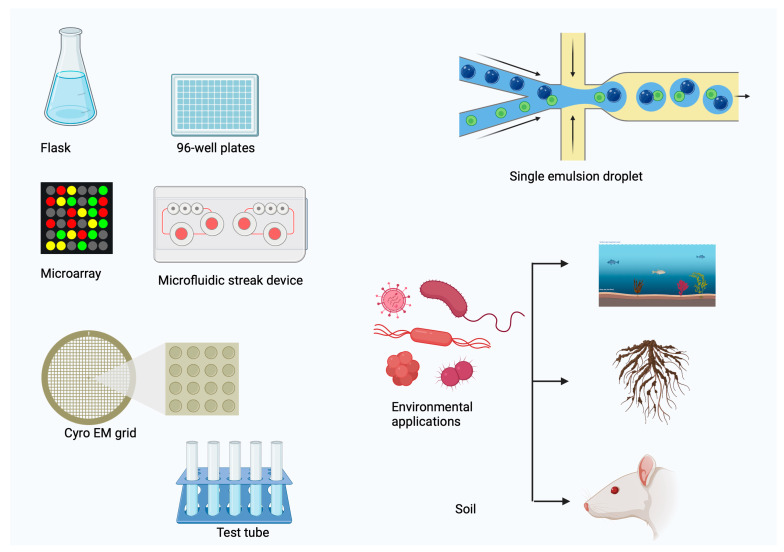
Schematic representation of new technologies against multiple resistant microbial strains.

Modern scientific research has prepared the way for developing synthetic antimicrobials by altering the chemical and structural makeup of naturally occurring goods to combat the rise of antibiotic-resistant bacteria. Novel antimicrobials have been fabricated and visualized using X-ray crystallography, structured-guided designs, and components based on primogenitor cell lines [18]. Oxepanoprolinamide is a derivative of lincosamide that has a higher therapeutic efficacy against resistant bacterial strains and an increased probability of overcoming multidrug resistance caused by ATP binding cassette (ABC) F-, Erm-, and Cfr genes [18]. Multiple resistant microbial strains have emerged recently, and new technologies have become available (Figure 1).

AMR is a key health issue in global health. It is based on more than just the many impacts on health; it also examines the economic and public policy dimensions outside the realm of health. The health implications of AMR are severe. As resistant infections increase, the load on healthcare systems also increases, resulting in increased morbidity and mortality. According to WHO estimates, if no action is taken, AMR could cause 10 million deaths every year by 2050. Hence, it would strain the health sector and cause many diseases to subjugate and resurface. Resistant pathogens are complicating the management of infectious diseases, challenging outbreaks of diseases like tuberculosis, HIV/AIDS, and malaria. In addition, AMR can erode all the gains made in the global health initiatives meant to reduce maternal and child mortality and improve surgical outcomes. The economic impacts of AMR are equally significant. The longer hospital stays, more expensive medications, and more diagnostic procedures raise healthcare costs. In addition, AMR will strangle economic growth because it influences labor productivity and increases the financial burden of healthcare. It also affects businesses that engage in agricultural and food production, which are at risk of being impacted by livestock health and food safety due to AMR, lowering farm productivity and increasing food prices. The resultant shocks on supply chains further destabilize economies and demonstrate how health and economic systems are interconnected.

There is a need for an effective public policy framework to push for coordinated action in many sectors to deal with AMR. Governments must prioritize health agendas, integrating AMR into national health policies and strategies. The public and health professionals have to be made aware of the responsible usage of antimicrobials. The other problem is the misuse and overuse of antibiotics in human medicines and agriculture. Effective management of AMR requires a One Health approach that recognizes human, animal, and environmental health inter-connectedness. It, therefore, involves policymakers seeking consultations with numerous stakeholders to establish holistic policies that recognize AMR on its multiple dimensions.

### 1.1. Mechanism of Antimicrobials

Antimicrobials are called agents that kill or inhibit the growth of microorganisms, including bacteria, fungi, viruses, and parasites. Depending on the target of action in the microbial cell, these agents can significantly differ in their mechanisms of action from one type to another or from one kind of antimicrobial to another. These can be grouped into several types: inhibition of cell wall synthesis, disruption of cell membrane function, interference with protein synthesis, inhibition of nucleic acid synthesis, and disruption of metabolic pathways. 

One of the best-known mechanisms is interference with cell wall synthesis. This is most notably with beta-lactam antibiotics, such as penicillins and cephalosporins. Here, the antibiotics act upon the PBPs that catalyze the cross-linking of peptidoglycan layers of the bacterial cell wall. Inhibition of PBPs weakens the cell wall structure, finally causing cell lysis and death, especially in rapidly dividing bacteria. This mechanism is effective, especially on Gram-positive bacteria with a thick peptidoglycan layer. Another mode of action is through cell membrane dysfunction. The integrity of the cell membrane is disrupted, and agents such as polymyxins cause the permeability to rise, resulting in death. It especially works on Gram-negative bacteria whose outer membrane is sensitive to antibiotics. The chemicals allow vital cellular contents to leak out from the compromised integrity of the membrane, hence killing the cells.

Protein synthesis inhibitors work by targeting the ribosomal machinery of bacteria. By binding to specific sites within the ribosome, protein synthesis inhibitors interfere with translation and prevent the synthesis of essential proteins for bacterial growth and reproduction. For example, in the case of tetracyclines, a tRNA molecule cannot be attached to the ribosomal acceptor site, while misreading of mRNA occurs on the part of aminoglycosides; as a result, some nonfunctional proteins are generated. Inhibitors of nucleic acid synthesis include fluoroquinolones and rifamycins. The agents are compounds that interfere with the processes needed to replicate DNA and transcribe RNA. Fluoroquinolones inhibit bacterial DNA gyrase and topoisomerase IV, enzymes that unwind DNA for replication. This interference with these enzymes leads to a cessation of bacterial growth. Rifamycins act by binding to bacterial RNA polymerase, thus inhibiting the production of RNA. Protein production is halted after that. Finally, some antimicrobials disrupt essential metabolic pathways of the microorganisms. Sulfonamides, for instance, inhibit folic acid biosynthesis by competing with PABA. Since most bacteria cannot obtain folate from their environment, their development is severely suppressed owing to the depletion of critical nucleic acids and amino acids.

### 1.2. Antimicrobial Activity of Uncultivated Microorganisms

A rich and largely untapped microbiology reserve is found in uncultivated microorganisms possessing antimicrobial activity. Many reside in diverse environments, such as soil, marine ecosystems, and extreme habitats, often possessing particular metabolic pathways that allow them to produce bioactive compounds. Soils are reservoirs of vast, unexplored microbial diversity, including bacteria and fungi, which might have antimicrobial activity. For instance, many bacteria from the Actinobacteria phylum, most belonging to the Streptomyces, have been reported for high antibiotic productivity. However, many close relatives of such microorganisms remain uncultivated in nature and, thus, may have new compounds for antimicrobial activity.

Some scientists have viewed marine sources of bacteria as the origin of potent bacteria with antimicrobial activities, such as Salinispora. As such, unique chemical environmental conditions of aquatic ecosystems influence the evolution of novel or new antimicrobial metabolites. Microorganisms that thrive in extreme conditions, such as those found in hot springs, salt flats, and deep-sea hydrothermal vents, often produce substances with antimicrobial properties as a survival mechanism. For example, the recently explored extremophile Thermus aquaticus has been shown to possess antibiotic properties, and some of its uncultivated relatives may provide new lead compounds with significant antimicrobial activity.

Identification of uncultivated microorganisms involves advanced techniques that combine molecular biology and bioinformatics. It can be started by collecting samples from ecosystems with high microbial diversity, such as soil, water, and extreme habitats. Samples will then be conserved carefully to preserve their microbial community. The DNAs of these microorganisms are sequenced at high throughput through various techniques, including, but not limited to, 16S rRNA gene sequencing or shotgun metagenomics after their extraction from the samples. This implies that there will be total coverage of the microbial community with all genetic contents considered of the cultivated and uncultivated microorganisms.

After sequencing, bioinformatics analysis is essential in the identification of uncultivated microorganisms. Using software tools such as QIIME(2023.7), Mothur(V1.45.3), or other alignment programs for sequences, researchers can classify the organism by analyzing these sequences. Phylogenetic relationships can be identified by relating the sequences to databases, thus linking a particular sequence to known genera or species, even though those organisms have not been cultured. More importantly, metagenomic data provide biosynthetic gene clusters that predict metabolic capabilities, including the possible production of antimicrobials. Computational approaches to uncovering these gene clusters also help understand the functional potential of uncultivated microorganisms. Single-cell genomics also helps to analyze individual cells directly isolated from the environment and can continue to aid in identifying and characterizing such organisms.

## 2. Bacterial Sources of Antimicrobials

Bacterial sources of antimicrobials have gained prominence in the search for new antibiotics, especially with the increasing resistance of microbes to antibiotics. Bacteria produce many bioactive compounds to survive in competitive environments, and many of these compounds possess antimicrobial properties. The most significant group of bacteria identified as antibiotic producers is the Actinobacteria, with the genus Streptomyces being the most important. These bacteria produce various peptides known as bacteriocins that have been reported to have activity against pathogens, including Gram-positive and Gram-negative bacteria. Besides *Streptomyces* and *Bacillus*, other bacterial genera like *Pseudomonas* and *Lactobacillus* are also important sources of antimicrobial activities. For instance, Pseudomonas aeruginosa produces several antimicrobial metabolites, including pyocyanin, which, besides having antibacterial activity, also plays a role in its virulence. Meanwhile, lactic acid bacteria from the genus *Lactobacillus* are best known for producing lactic acid and other antimicrobial compounds that inhibit food spoilage organisms and pathogens; therefore, their importance is amplified in food safety and preservation.

Bacteriocin, organic acids, diacetyl, hydrogen peroxide, and other antimicrobials are produced by lactic acid bacteria (LAB). However, different bacteria also produce many, not exclusively by LAB. While the most famous producers of bacteriocins belong to LAB species, for example, Lactobacillus and Lactococcus, some E. coli strains can produce bacteriocins. The growth of some bacteria can be inhibited by the bacteriocin produced by the boza-isolated *Lactobacillus pentosus* ST712BZ [19,20]. Bacteriocin was discovered by Andre Gratias in 1925 when he realized that colicin V is a bacteriocin produced by a subset of E. coli strains that target and inhibit close relatives of bacterial species. This wide number of bacteriocin producers indicates that bacteria possess the mechanisms to compete competitively in different environments, including the gut and food products since they are more opportunistic [21].

Hernández-González categorized bacteriocins based on their molecular weight, susceptibility to enzymes, capacity to withstand high temperatures, presence of modified amino acids after translation, and mechanism of action [22]. Class I separates lantibiotics into Ia and Ib by chemical structure and charge. Bacteriocin classes IIa, IIb, and IIc exist [22]. Some heat-stable peptides weigh less than ten kDa. Class IV consists of large peptides and carbohydrates or lipids, whereas Class III includes thermo-labile peptides with a high molecular weight, often exceeding 30 kDa, such as Helveticin J [23]. Despite competing theories by researchers such as Cotter et al. [24] and Drider et al. [25], there is still no one-size-fits-all solution.

There are currently four main categories into which bacteriocins are classified based on their biochemical and genetic characteristics [25]. Mechanisms of bacteriocins go beyond the Class IIa bacteriocins of LAB, such as lactococcin A. Indeed, while Class IIa bacteriocins often kill their target by making pores in the cell membrane and lysing the target, other bacteriocins have mechanisms that vary and contribute to their properties. Class I bacteriocins, known as lantibiotics, are characterized by post-translational modifications that introduce unusual amino acids. One such compound is nisin, which can interfere with cell wall synthesis and cause pores in the membranes of susceptible bacteria. Besides causing membrane damage, lantibiotics can inhibit peptidoglycan biosynthesis, compromising the structural integrity of the bacterial cell. This makes them doubly effective against Gram-positive bacteria. Class IIb bacteriocins require two peptides to be effective against their target, typically synergistically to act on the cell membrane of the target cell. Like Class IIa bacteriocins, Class IIb bacteriocins are thought to disrupt the target cell membrane. The two peptides might increase the efficacy and broaden the spectrum of activity of Class IIb bacteriocins. This mechanism of action provides a broader scope for inhibiting various bacteria. Class III bacteriocins are distinct from the other two classes because they are larger, heat-labile proteins with enzymatic properties. These bacteriocins can degrade important components of the bacterial cell, such as peptidoglycan, inhibiting growth without forming pores. Their enzymatic activity can target structures within the bacterial cell for an alternative action that may be effective against resistant strains. Class IV bacteriocins are composite molecules bearing additional nonpeptide parts such as carbohydrates or lipids. Extra components given by such portions confer antimicrobial activity that can be mediated in terms of cell membrane disruption or interfering with metabolic pathways. However, the mode of Class IV bacteriocins varies immensely, though every mechanism offers new methods against bacterial growth.

The antimicrobial effect of bacteriocins is determined by their main structure [26]. Some bacteriocins enter cells and interfere with protein synthesis and gene expression [26], while others interfere with the proton motive force of electrified membrane vesicles [27]. Antibiotics have a dual action against bacteria [26,28,29]. Table 1 shows different bacteriocins and their producers for resistance in bacteria.

When non-lantibiotics bind to the mannose phosphotransferase permease (Man-PTS) subunits C and D, they establish an intra-membrane channel that allows ions to move, ultimately leading to cell death [29]. Because of their high net positive charges, circular bacteriocins electrostatically attract bacterial membranes that are negatively charged [30]. Cell death, alterations to the membrane potential, ion efflux, and holes are all results of this interaction [31]. The cell wall hydrolyzes more quickly due to bacteriolysins, which gradually causes the cell to disintegrate [32,33]. Target cells’ ability to take up glucose is inhibited by bacteriolysis, leading to their death from starvation [34,35,36].

Antibiotics are essential antimicrobial agents that have revolutionized the treatment of bacterial infections since their discovery. There are many classes of antibiotics, and each class has a unique mechanism of action. For instance, penicillins and cephalosporins inhibit cell wall synthesis, eventually leading to cell lysis. Though antibiotics have been very effective, several challenges have been observed. In human medicine, agriculture, and livestock, antibiotics have been overused and misused, which gave rise to resistant bacterial strains. This complicates the treatment options and leads to protracted illness, higher healthcare costs, and deaths. In this regard, the medical community focuses on antibiotic stewardship; it promotes responsible use of antibiotics in order not to develop resistance. This is achieved by research in developing new antibiotics and alternative therapies against infections. This includes explorations into bacteriophage therapies and viruses that selectively infect bacteria. The mechanism of action and antibiotic resistance can provide guidelines for developing and creating new strategies to maintain efficacy and develop new treatments for bacterial infections.

Synergistic or antagonistic interactions can occur between antimicrobial drugs and the microorganisms they are effective against [37]. Nisin and other bacteriocins kill *E. faecalis* and other bacteria that cause periodontal disease, preventing gingivitis and antibacterial plaque in dogs [26]. Some *Pseudomonas* species produce the anionic biosurfactant rhamnolipid [38,39]. Antimicrobial, antibiofilm, antitumor, and antioxidative properties have all been observed for these compounds [40,41,42].

The interaction of the antimicrobial drug with the targeted microorganism determines the treatment outcomes, thus leading to strategies for combating infection. Synergistic interaction describes an interaction between two or more antimicrobial agents to synergistically enhance their total effect, creating a final treatment outcome more efficient than if such effects had occurred alone. One such example that demonstrates the synergistic effect is the therapy of infections caused by Enterococcus faecalis involving the administration of penicillin simultaneously with aminoglycosides, including gentamicin. Aminoglycoside penetration and action on the protein synthesis process can occur since the bacteria cell wall is destroyed with the introduction of penicillin. The potentiation of different drugs could effectively achieve better outcomes in treating a serious infection caused by resistance-sensitive microorganisms. Another example is the use of sulfonamides and trimethoprim; together, they inhibit far more significantly than either medication alone the bacterial synthesis of folic acid. Again, this synergism occurs very commonly in patients diagnosed with urinary tract infections or infections of certain types of pneumonia and shows how combining these medications can increase efficiency, possibly at a reduced risk of resistance.

Conversely, antagonistic interactions are noted when one antimicrobial agent decreases the effectiveness of another and hence produces a lesser therapeutic result. For instance, when combined with bacteriostatic agents like tetracyclines, bactericidal antibiotics such as Penicillins produce antagonistic interactions. The bacteriostatic effect of tetracyclines leads to the retardation of bacterial growth, and this may interfere with the bactericidal action of antibiotics that depend on the active development of cells for the lethal effect. This could subsequently lead to treatment failure for a few patients with these bacteria, which should be removed immediately since the patients have severe infections. Implications of these interactions are the synergistic combinations that would allow better efficacy of the two drugs used in a given treatment, reduced dosages required by the patients who may need the drugs at reduced levels, and alleviating the associated side effects, thereby providing better outcomes. This also helps combat antibiotic resistance because lower dosages of antibiotics reduce selective pressure, thereby reducing the development of resistance.

They can modify viral membrane glycoproteins and interact with viral lipid membranes, especially in light of their use in combating bovine coronaviruses, HSV-1 and HSV-2 [43,44]. The rhamnolipids (M15RL) produced by the Antarctic bacterium *Pseudomonas gessardii* (*P. gessardii*) M15 have been shown to inhibit SARS-CoV-2 [45].

**Table 1:** Bacteriocins and their producers for resistance in bacteria.

Bacteriocin	Bacteriocin Producer	Reference(s)
Nisin A	*Lactococcus lactic* subsp. *lactis*	[46,47,48]
Nisin ANisin V	*L. lactis*	[49]
Pediocin A	*Pediococcus pentosaceus FBB61*	[50]
Enterocin M	*Enterococcus faecium AL41*	[51]
Enterocin CLE34	*Enterococcus faecium CLE34*	[52]
Enterocin E-760	*Enterococcus faecium*, *Enterococcus durans*, *Enterococcus hirae*	[53]
Lacticin 3147	*Lactococcus lactis*	[54]
Macedocin ST91KM	*Streptococcus gallolyticus*	[55]

## 3. Bacterial Sources of Antifungal Compounds

Bacterial sources of antifungal compounds refer to bacteria that naturally produce substances that can inhibit or kill fungal pathogens. These bacteria synthesize bioactive molecules, including peptides, polyketides, alkaloids, and other secondary metabolites, identified as effective antifungal agents. Many of these bacterial strains, particularly those from the Streptomyces genus and other actinobacteria, are known for their potential to combat fungal infections that pose serious threats to human health, agriculture, and food preservation. Exploring bacterial sources for antifungal compounds is critical, as it provides an alternative to traditional antifungal treatments, helping address the growing issue of antifungal resistance. These natural products are increasingly being studied for their unique mechanisms of action and potential in developing new antifungal therapies. The red pradimicins A, B, and C are produced by *Actinomadura hibisca* [56]. These pradimicins are effective against Aspergillus and Candida [57] [Table 2]. Pratimimins are benzonapthacenequinones that transport D-alanine and carbohydrates, as revealed by spectral analysis and chemical breakdown [56]. By forming a compound with the cell walls of vulnerable microbes, pradimicins damage fungal cells by binding to terminal D mannosides [57]. Many species of *Actinoplanes* create antifungal metabolites as well.

Purpuromycin, produced by *Actinoplanes ianthinogenes*, is effective against *Trichophyton mentagrophytes* [58]. Streptomyces species produce branching bacilli that are aerobic and Gram-positive bacteria. The chemicals nystatin, phoslatomycins, phthoxazolin A, faeriefungin, butyrolactols, sultriecin, polyoxin, and dunaimycins are examples.

Aspergillus species, Saccharomyces spp., and *Candida albicans* can be effectively targeted by azobacilin, bacereutin, cispentacin, and mycocerein, which are generated from *Bacillus cereus* compounds, as stated by Kerr [58]. *Bacillus licheniformis* produces both the peptide A12-C and fungicin M-4 [63,64]. According to research by Chernin et al., *Enterobacter agglomerans* become antibacterial against *Aspergillus niger*, *Candida species*, phytopathogenic fungi, and dermatophytes when treated with pyrrolnitrin.

A and B, two antifungal herbicolins, are again produced by *Enterobacter agglomerans* [65,66,67]. The dipeptide CB-25-1 from *Serratia plymuthica* [68], Dihydroaeruginoic acid [69], pyocyanin [70], and 1-hydroxyphenazine [71] are compounds created by *P. aeruginosa*, which is present in a healthy human stomach. Pseudomonas also produces the peptide family pseudomycin [72], the caryoynencins [73], and the cyclic hydroxamic acid, G1549 [74], among other antibacterial chemicals.

Antimicrobial chemicals and bacteria can also be found in Burkholderia species [75]. Cepalycin [76], xylocaine [77], and heptylmethylquinolinone [78] are some of the other compounds that *B. cepacia* can create. The antibacterial enacyloxcins are thought to have originated in the Burkholderia species [79]. There are eight different antibacterial chemicals in the enacyloxcin family [76,77]. Maltophilin gives the Rhizosphere strain of *Stenotrophomonas maltophilia* antifungal characteristics. Antifungal action against *Neurospora crassa*, but not yeast, has been observed for polyenic antibiotics from the genus Gluconobacter [80].

## 4. Fungal Sources of Antimicrobials

Since the isolation of penicillin G from a fungal species in 1928 [81], fungi have emerged as promising candidates for developing new antimicrobial drugs due to their prolific secondary metabolite production. Enfumafungin, a triterpenoid extracted from a Hormonema species, was first identified more than ten years ago and has since been demonstrated to be particularly efficient against *Candida* spp. and *Aspergillus* spp. [82]. Enfumafungin is a new antifungal agent that inhibits glucan synthesis in the fungal cell wall by inhibiting the enzyme β-1,3-glucan synthase. The drug binds to the active site of this enzyme so that the enzyme cannot catalyze the formation of β-1,3-glucan, a crucial component of the fungal cell wall. This inhibition weakens the structural strength of the cell wall of the fungi, thereby making their cells susceptible to osmotic pressure and then lysing them. A primary advantage of enfumafungin is that its toxicity is selective; mammals do not synthesize β-1,3-glucan, meaning the compound has less of a chance of causing harmful side effects by negatively impacting human cells. With its broad-spectrum antifungal activity spectrum against most pathogens, namely Candida and Aspergillus species, enfumafungin makes the ideal drug for treatment protocols in cases of invasive infection among immunocompromised patients. Its novel action mechanism combined with synergy offered when added to other known antifungal agents justifies this therapeutic profile it presents within the battle toward resistance at the clinic stage. Phase II clinical trials are underway for SCY-078, a semisynthetic derivative of enfumafungin [83,84].

However, in recent years, favolon, a metabolite of strobilurins generated by *Favolaschia calocera* (*F. calocera*), has been found and demonstrated to be less toxic while still displaying substantial antifungal action against human pathogens [85]. By disrupting quorum sensing, fungal metabolites prevent biofilm formation. Coprinuslactone, isolated from *C. comatus*, inhibits *P. aeruginosa* biofilm formation [86]. Quorum sensing is a complex communication mechanism bacteria use to coordinate their behavior with population density. This is achieved by producing and releasing signaling molecules known as autoinducers. The concentration of these autoinducers increases with an increase in the population size of the bacteria. At a certain threshold concentration, bacteria detect these molecules through specific receptors, initiating a series of intracellular signaling pathways. It will lead to coordinated changes in behavior, such as the formation of biofilm, virulence factor production, and sporulation. For instance, Pseudomonas aeruginosa utilizes quorum sensing to manage its virulence factors by controlling them at a point that maximizes its pathogenicity when it grows in high-density populations. The ecological relevance of quorum sensing is its effects on microbial community dynamics and interaction between species, allowing the bacteria to adapt to changes in their environment. Clinically, it is helpful because it contributes to the pathogenicity of many bacteria. It opens up novel therapeutic approaches: quorum sensing inhibitors or “quorum quenching” agents that interfere with bacterial communication, which reduces virulence and may offer alternatives to traditional antibiotics. A Kenyan basidiomycete’s microporenic acid A is lethal to biofilms of *Staphylococcus aureus* and *Candida albicans* [87]. Antibiotics that also work against biofilms hold great promise.

## 5. Viral Sources of Antimicrobials

An interesting area of research that includes the viral source of antimicrobials is the development of new therapeutic agents that can counter antibiotic-resistant bacteria and other pathogens. The bacteriophages or phages are viruses specific to bacteria and are considered a promising source of antimicrobial compounds. These viruses can efficiently target and lyse bacterial cells, making them useful in clinical and agricultural applications. The best-studied viral origins of antimicrobials include bacteriophages, which can target specific bacterial strains and are less harmful to the microbiota. Because of such specificity, treatment can be directed to maximize the antimicrobial effects while keeping the detrimental impact on normal flora minimal. Phages exert their antimicrobial effects through several mechanisms. When a phage infects a bacterial cell, it injects its genetic material, hijacking the host cellular machinery to replicate itself. This leads to the formation of new phage particles; this causes the lysis of the bacterial cell and the release of these new viruses into the surroundings. Some phages produce endolysins, enzymes that degrade the cell walls of bacteria for further killing of bacteria. Other areas of interest include viral proteins, such as lysins from bacteriophages, which can be applied topically or systemically to target specific bacteria selectively. Lysins may find applications in removing biofilms, where conventional antibiotics are difficult to use.

Influenza Rubylide S, produced by the marine fungus *Aspergillus terreus* OUCMDZ-1925, inhibited the activity of the H1N1 virus [88]. *Cladosporium sphaerospermum* 2005-01-E3 yielded a new hybrid polyketide called Cladosin C. *Penicillium chrysogenum* PJX-17 is the source of the potent H1N1-fighting sorbicillinoids sorbicatechols A and B [89].

Trypilepyrazinol has demonstrated antiviral activity against several viruses, including HIV and HCV [90]. Aspernigrin C and malformin C, which *A. niger* SCSIO Jcsw6F30 produced, had strong antiviral activity against HIV-1 [91]. The *Streptomyces kaviengensis* (F7E2f) isolate antimycin A blocks mitochondrial electron transport and pyrimidine synthesis, making it effective against WEEV [92] [Table 3].

## 6. Antimicrobial Peptides

Various naturally occurring compounds known as antimicrobial peptides (AMPs) protect hosts from pathogens, including bacteria, fungi, parasites, and viruses [100]. Small in size, most AMPs are cationic because they contain several lysine or arginine residues. This positive charge allows AMPs to interact with the predominantly negative bacteria membranes. However, some AMPs have anionic properties [101,102].

The synergistic action with traditional antibiotics, low resistance risk, low efficacy at low concentrations, and broad-spectrum AMP activity of AMPs have attracted attention [103,104]. They act on multiple plasma membrane and intracellular targets to destroy pathogenic organisms. AMPs are antibacterial, antifungal, antiviral, antiparasitic, and immunomodulatory [100]. AMPs are different molecules that attack many targets in microbial cells, mainly the plasma membrane and intracellular components. The main mode of action for most AMPs involves disruption in the lipid bilayer’s permeability from the microbial membranes, eventually killing cells by creating pores or channels, leading to leakages of crucial ions and molecules. This disruption of the membrane is more common in cationic peptides, for instance, defensins and cathelicidins that show a high preference towards binding certain lipid components of a higher abundance in the bacterial membrane than in eukaryotic cells, thereby causing less toxicity in the host cells. More than that, some AMPs could penetrate the plasma membrane and target the processes inside the cell. Here, they may inhibit certain metabolic or cell wall-synthesizing enzymes. They can also interact with proteins, thus blocking crucial functions like protein synthesis or signaling pathways. Other AMPs may interact with nucleic acids, which then block the replication and transcription of DNA or RNA. This multi-step mechanism of action against the microbial cells is responsible for the broad-spectrum activities of AMPs against pathogens, including bacteria, fungi, and viruses, making them in the front run for new antimicrobial therapies. While treating patients with VRE, Acinetobacter baumannii, or MRSA, *Staphylococcus aureus*, *Listeria monocytogenes*, and the bacterium *E. Foodborne E. coli*, *Salmonella*, and *Vibrio parahaemolyticus* infections can be prevented with the use of AMPs.

Defensins, nisin, and cecropins are AMPs that kill Gram-positive and Gram-negative bacteria. Both the *Aristicluthys nobilia* interferon-I inspired AMP P5 (YIRKIRRFFKKLKKILKK-NH2) and AMP P9 (SYERKINRHFKTLKKNLKKK-NH2) have been demonstrated to inhibit MRSA [105]. As a result of AMR, there has been a dramatic increase in demand for AMPs, which are used in both human healthcare and agricultural settings.

## 7. Antiviral Peptides

Viral illnesses have been documented ever since recorded history began. Scientists began isolating viruses in the nineteenth century. Viruses are still a leading source of human disease, possibly related to the lengthy process of finding and developing new vaccines [106]. Although antiviral medication is being used, it is not without risk. Because viral epidemics like H1N1, Ebola, and Zika develop and reappear quickly, several antivirals have little efficacy. Such a statement refers to the problems some antiviral drugs face during viral outbreaks. A good example is what occurred with H1N1, Ebola, and Zika viruses. Oseltamivir (Tamiflu) is ineffective against H1N1 viruses in certain outbreaks because they develop certain mutations that make them resistant. The traditional antivirals for other viruses may not work against those that evolve at a great speed, like Ebola and Zika, which can accelerate the epidemic’s speed much faster than specific treatments can develop and become effective. Furthermore, the narrow therapeutic window of some antivirals, coupled with the urgent need for intervention, brings into sharp focus the weakness of the current antiviral therapies in dealing with emerging and re-emerging viral threats. This only emphasizes the need for constant research and the development of new antiviral drugs that can target a more extensive range of viruses or utilize alternative mechanisms of action to offer effective treatment at such times of outbreak. There is a growing demand for more effective antiviral medications. Antiviral peptides (AVPs) have been studied extensively recently as a potential first line of defense against viruses [106]. These are small, naturally occurring, or synthetic molecules that interfere with the viral infection at any stage by binding to viral components, altering host cell processes, and so on. Many mechanisms of action of such peptides are based on interference with the viral entry; indeed, several antiviral peptides can bind viral envelope proteins and thus interfere with their interaction with the receptors on the host cells. For example, the HIV-derived peptide inhibits the entry of the virus by binding to its envelope proteins. Beyond this action of inhibiting virus penetration, antiviral peptides can enhance host immunity. This can be achieved by stimulating the host’s immune cells and the subsequent production of cytokines. Such immunomodulatory activity will contribute more to an effective immune response against the viruses invading a host. In some other respects, other peptide actions can affect the replication process when they become attached to the viral proteins or nucleic acids upon having entered the host cell. These peptides inhibit virus replication by inhibiting core enzymes like polymerases and proteases. Unlike classical antiviral drugs, antiviral peptides exhibit quite impressive advantages. They frequently have multiple targets against diverse viruses and are less susceptible to developing resistance because these peptides’ mechanisms of action are quite different. Moreover, the action of antiviral peptides is rapid as these can act to prevent or abort entry or replication. Thus, the time elapsed due to infection is lessened as well. On the other hand, this method’s challenges are stability, bioavailability, and toxicity. Traditional antiviral drugs generally have more favorable pharmacokinetic profiles, whereas peptides might be less stable and must be delivered using specialized drug delivery systems. Syntheses of antiviral peptides may also be more complex and expensive, which could impact access and clinical uses.

One AVP, clavain, is sourced from the tunicate *Styela clava* [107]. Viruses other than HIV have been inhibited by clavanin A and clavanin B, respectively [108,109]. Some HIV drugs include enfuvirtiude, derma septin-S1, S4, dermaseptin-S2, magainin 2, dermaseptin-LL-37, Carin 1, maximin 3, gramicidin D, and siamycin-I and -II [110]. Antiviral peptides against the coronavirus are being developed in response to the recent COVID-19 epidemic.

## 8. Climate and Terrestrial Changes in Microbes

Phytoplankton in the oceans reproduce far more quickly than tree species (days vs. decades), are more distributed, and are less affected by seasonal shifts. Phytoplankton are among the first organisms that respond rapidly to climate shifts [111,112,113,114,115,116]. A general tree species characteristic is long life cycles and slower reproduction rates. Some of these take decades to achieve maturity or bear seeds. They are usually less mobile; distribution mainly depends on the soil type, climate, and ecologic interactions. Although trees produce much biomass and stabilize the ecosystem, their slower growth and reproduction rates make them incapable of changing populations rapidly compared with the agile phytoplankton in the oceans. This basic difference explains how these two groups of organisms adjust to their respective environments and ecological roles. Chemolithoautotrophic bacteria and archaea fix CO_2_ and supplement the role of marine phytoplankton in CO_2_ sequestration [117,118,119] in both deep-sea waters [120] and surface waters in the polar winter [121]. Chemolithoautotrophic bacteria are autotrophic microorganisms that obtain energy from oxidizing inorganic compounds, including hydrogen sulfide, ammonia, ferrous iron, and hydrogen gas through carbon dioxide, the major carbon source for their growth. This metabolic mechanism allows for survival in habitats with very scarce organic material, like deep-sea hydrothermal vents and natural sulfur springs; hence, these organisms play the most integral role in primary production within these environments. The photosynthetic activities of making CO_2_ turn into carbon-based organic substances through chemical reactions, such as in the Calvin cycle, build the backbone of the food chain, thus supporting other biological communities within the habitat. They also play an important role in biogeochemical cycles by oxidizing ammonia and producing nitrite and nitrate in the nitrogen cycle; they also play a significant role in the sulfur cycle. Other applications of these bacteria lie in biotechnology and the environment, such as bioremediation and producing bioenergies. Its specificity means it is ecologically and practically useful in solving or preventing possible environmental problems.

Global biogeochemical cycles [122] are affected by climate change because of its effect on predator-prey relationships, particularly virus-host interactions. Dissemination is typically simpler for bacteria than it is for larger creatures. Many microbial species vary biogeographically due to dispersal, lifestyle (including the influence of host association), and environmental variables that impact the composition and functioning of communities [123,124,125,126]. Temperature and latitudinal gradients are particularly significant for marine ecosystems [127,128]. Ocean acidification and other environmental variables related to climate change have received very little attention in evolutionary studies [129,130]. Little is known about the molecular mechanisms underlying physiological reactions or their biogeochemical consequences [131].

Land microorganism numbers are about 10^29^, like aquatic ones. Soil bacteria regulate the amount of organic carbon kept in soil and released into the atmosphere [132,133,134], in addition to controlling productivity with macronutrients like phosphate and nitrogen. Mycorrhizal fungi help plants obtain nitrogen and phosphate in various environments [134]. Plants provide their fungal symbionts with lots of carbon.

Up to 25% of all man-made carbon dioxide (CO_2_) is stored in forests [135,136], and forests account for 50% of all terrestrial primary productivity. Grasslands account for about 29% of Earth’s surface area. These dry and semiarid regions respond differently to human-caused climate change than their forested counterparts [137,138], and they are crucial to the carbon budget. Lakes comprise around 4% of the Earth’s non-glaciated surface area, and many methane gas emissions come from shallow lakes [139,140].

Climate change affects microbial community structure and diversity through seasonality, temperature, plant composition, litter, and root exudates [141,142]. Natural geothermal warming in the lab and the long run (more than 50 years) boosted the respiration and biomass of soil microbes, which released net carbon dioxide. At the same time, substrates were depleted, decreasing biomass and microbial activity [143]. Hence, it is clear that some microbial groups find it difficult to adjust to warmer environments.

**Figure 2:**
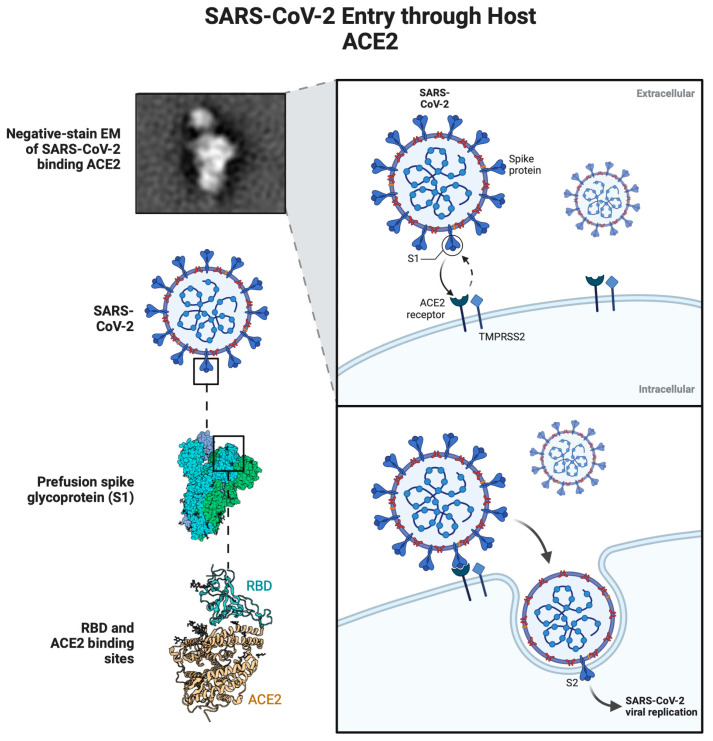
Antiviral peptide P9, produced from mouse β-defensin, inhibits SARS-CoV-2 infection by binding to viral glycoprotein and masking host cell ACE2 receptors.

Climate change impacts several environmental characteristics [144], which impacts illnesses in marine and terrestrial organisms [145] (Figure 2). Modifying their defensive systems and nutrient cycling routes may make corals more vulnerable to bleaching and disease [146] due to changes in their microbiome brought about by warming oceans. Damage to fish tissue from ocean acidification may impair their immune systems, allowing germs to colonize their bodies more easily [147]. Pathogens [148] potentially contribute to forest mortality due to drought and heat stress.

As a result of changes in host and parasite acclimation [149], climate change can raise disease risk. Temperature might increase susceptibility to infection for ectotherms (like amphibians), potentially by perturbing immune responses [149,150]. The Cuban tree frog is more vulnerable to Batrachochytrium dendrobatidis when temperatures fluctuate weekly and daily. Assessing host-pathogen reactions directly is of utmost importance rather than making assumptions based on research solely focused on the growth rates of individual microorganisms to determine the significance of climate change accurately [149], as there is a discrepancy between the effect of rising temperatures on infection and the fungus’ reduced growth capacity in pure culture. Recent research indicates that antibiotic resistance among human infections is projected to increase due to climate change [151]. Antibiotic resistance in human infection will surge due to global warming caused by complicated and intricate environmental, biological, and social interactions. Increasingly rising temperature levels worldwide are likely to shift significantly in pathogen-carried genes relating to resistance, resulting in the prevalence of antibiotic-resistant microbes affecting populations. When the temperature is warmer, microbe metabolism increases in organisms. As a result, a higher chance of mutation occurs, and correspondingly, a higher possible rate of developing resistance-based traits arises. Climate change disturbs the balance of some microbial communities in different parts of the ecosystem, thereby letting ARBs outcompete susceptible strains. More instances of antibiotic resistance may also pose significant challenges to the capability of public health systems to control the increasing tide of resistant infections. Such infections usually require an extended stay in a hospital, which naturally increases medical costs. The patients most adversely affected are the elderly, including the immunocompromised. In addition, interconnectivity in our globalized world has made things worse. With trade and travel, antibiotic-resistant bacteria can spread quickly across borders. Climate change will increase the occurrence of natural disasters, displacing the population and crowding places where resistant infections thrive well. Climate-induced migration further introduces resistant strains into a new environment, complicating local public health efforts and responses. Due to climate change, the transmission season may last longer, the rate at which diseases replicate in the vector may speed up, and the number and distribution of mosquitoes may grow. El Nio/Southern Oscillation (ENSO) and other large-scale climate phenomena significantly impact the spread of many infectious diseases, especially in vector-borne or aquatic environments. ENSO is associated with several significant human and animal illnesses, including malaria, dengue fever, Zika virus disease, cholera, the plague, African horse sickness, and many more [152,153,154,155,156].

## 9. Tools and Techniques

The tools and techniques used for identifying new microorganism-based antimicrobial drugs and finding new bioactive chemicals effective against disease-causing microorganisms are important because of the spread of antimicrobial resistance. The challenges encountered over the years include the inherent low concentrations of antimicrobials in natural sources, the absence of advanced and inventive drug discovery tools, and the substantial costs and limited investment in drug development and discovery [157,158]. The following are examples of conventional methods for extracting useful chemicals from bacteria:

- Examples of diffusion processes include Agar discs, antimicrobial gradients, wells, plugs, cross streaks, and poisoned food. The agar disc diffusion method was created in 1940 to determine bacterial resistance [159]. Detecting some bacterial infections is difficult [160]. This comprises *Streptococci*, *H. influenzae*, *N. gonorrhoea*, *N. meningitidis*, and *H. parainfluenzae*. Agar-grown bacteria are exposed to a predefined dosage of the test chemical in this assay. The test compound’s antimicrobial properties permeate into the agar, stopping the growth of sensitive bacteria. The zone’s diameter is measured when a growth inhibitor is used [161,162]. Agar disc diffusion is a simple and cost-effective method for determining the minimum inhibitory concentration (MIC). Nevertheless, the outcomes are imprecise [161]. The agar disc diffusion method is generally known to be a simple and cheap technique to determine the susceptibility of microorganisms to antibiotics. This is usually applied in clinical microbiology laboratories. This method, though easy to use and affordable, has inherent limitations that might affect the accuracy of the results. The important thing about agar disc diffusion is that it measures the diameters of inhibition zones developed around discs impregnated with antibiotics, and the resulting data are not quantitative measurements but rather qualitative. A qualitative nature in such tests leads to the possibility of getting vague estimations for the MIC—that is, the lowest concentration of an antibiotic at which visible growth of microorganisms is inhibited. Factors such as agar depth, inoculum density, and diffusion characteristics of the antibiotic may also contribute to variability in inhibition zone interpretation, which could then lead to misclassifications of resistance or susceptibility. Therefore, although the agar disc diffusion method is a good preliminary screening tool, it should be complemented with more quantitative methods, like broth microdilution or automated systems, to provide an accurate MIC and antimicrobial susceptibility determination;
- The antimicrobial gradient technique (Etest) may be utilized to calculate the MIC of antibacterials, antifungals, and other antimicrobials, which combines dilution and diffusion techniques. This technique can also analyze medication interactions [161,163,164,165]. The Etest, alternatively known as the “kilometer test,” represents a semi-quantitative method that shares the characteristics of the agar disc diffusion method and broth microdilution. This method leaves a plastic strip coated with a gradient of antibiotic concentrations on an agar plate seeded with the microorganism of interest. Once placed in the incubator for incubation, the antibiotic begins to diffuse into the agar, creating a concentration gradient. Microorganisms grow in the zone with inhibition around the antibiotic strip, and the interphase zone where the microorganisms grow on the bacteria is the MIC of an antibiotic. Etest’s main advantage lies in being a simple procedure that brings out quantitative results in just one step without the equipment being complex, such as in determining the MICs. It can be used for a wide range of bacteria and fungi and is effective for organisms that are difficult to culture or that exhibit slow growth. However, although the Etest is more precise than the agar disc diffusion method, it still has limitations related to variability in results that are influenced by factors such as inoculum density and agar composition;
- Depending on the analysis type, broth or agar dilution might be utilized. Typically, agar dilutions are used to investigate the efficiency of combinations of antifungal pharmacological drugs against *Candida* sp., *Aspergillus*, Fusarium, and dermatophytes [166,167];
- Several methods exist for screening and determining the susceptibility of microorganisms to antimicrobial medications. These include the time-kill test [168], the ATP bioluminescence assay [169,170,171,172], and the flow-cytofluorometric approach [173]. Time-kill tests refer to quantitatively determining antibacterial activity that compares time as a measure over some interval. In practice, it involves a uniform inoculum of a specified microbial isolate exposed to the established concentration of the specific drug under study within broth culture at a controlled number of hours or days in one series of studies that include the collection of specimens to obtain data on survival to perform subsequent plating to ascertain colonies. Time-kill tests are assessed as log reductions of the number of bacteria compared to the inoculum. The method may illustrate bactericidal and bacteriostatic action by showing how soon and effectively an agent kills bacteria. The time-kill test is useful for determining the effectiveness of combination therapy and discern dynamics regarding resistance development. ATP bioluminescence has allowed for the evaluation of cellular ATP levels. The luciferin–luciferase bioluminescent test is popular due to its high sensitivity. A high quantum yield chemiluminescent reaction with MgATP^2+^ oxidizes luciferin catalytically via the luciferase. Light intensity correlates with ATP levels under ideal conditions. Stimulating ATP release from a disintegrating cell to react with luciferin–luciferase and produce light can determine cellular ATP. A luminometer is used to quantify the brightness of an object;
- A flow cytometer and flow cytofluorometric approach can identify antimicrobial resistance and predict the chemical’s effect on microbe cell damage and viability [174].

## 10. Advances in Micro-Culturing Technology

**Figure 3:**
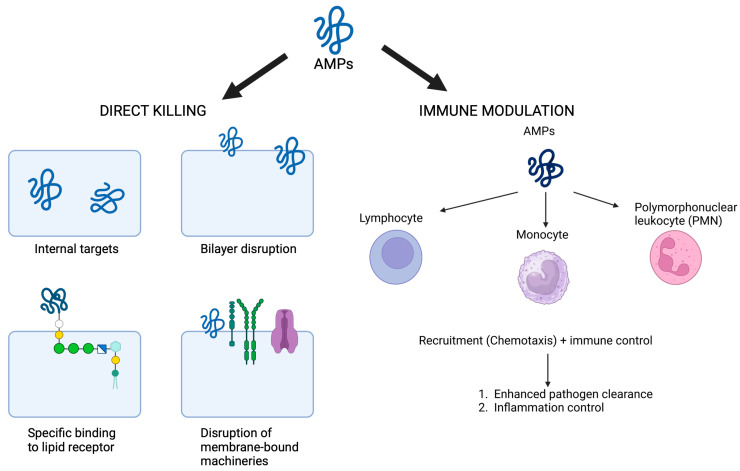
Mechanism of action of antimicrobial peptides.

Important equipment in laboratory practices, such as microwell plates, are also widely used for high-throughput screenings, assays, and experiments requiring accurate and reproducible conditions for sample analysis. In 1951, Hungarian scientist Dr. Gyula Takatsy invented microwell plates, the first to minimize laboratory sample sizes. Figure 3 from Banks [94] illustrates how his idea completely changed laboratory titrations and serial dilutions. There are several drawbacks to using 96-well culture plates. It is not genuinely high-throughput, and evaporation makes it difficult to keep a constant volume in a culture over time. Furthermore, waste product accumulation within wells and restrictions in oxygen transmission restrict the expansion of confluent cells. Finally, Ingham et al. [95] point out that the low picture resolution of microwell plates makes it hard to visualize qualitative data.

The complicated interactions between microorganisms and high-throughput screening required 50 years of “micro-culturing” progression. Miniaturizing culture conditions allows for the isolation and compartmentalization of microorganisms. Fast-growing species often obscure slower-growing ones due to this type of competition. However, although they are relatively rare, slow-growing organisms account for a sizable fraction of microbial diversity [96]. Slow-growing microbes constitute a large fraction of the microbial diversity, largely restricted to soil, aquatic, and human microbiome niches. An example would be the genus Mycobacterium, for example, *Mycobacterium tuberculosis*, which is slow growing. They can have generation times in days or weeks but play important roles in either disease or environmental processes. Another example is the genus Bacteroides, predominant in the human gut microbiome. Although some species within this genus grow rather easily, many are classified as slow-growing and play crucial roles in digesting complex carbohydrates and general intestinal health. Many species of the phylum Actinobacteria, especially from the genus Streptomyces, are slow-growing but highly productive regarding antibiotics and other bioactive compounds, highlighting their ecological and pharmaceutical importance. These slow-growing organisms are hard to culture in the lab but represent a considerable fraction of microbial diversity, significantly influencing environmental interactions and human health. Microenvironments are highly understudied, and the microtechnologies discussed here allow researchers to do so on spatiotemporal scales relevant to microorganisms [97].

## 11. Microarrays

Cell culture transitioning from milliliter scale to microliter scale starts with the technology of microarray, which includes automation and manual methodology developed to deposit cell droplets in a liquid or an alginate gel onto microscope slides. The microarray has several possible uses, including, but not limited to, studying phenotypic, morphological, proliferative, and cell-cell communication outcomes of cell-culture miniaturization [98].

The “nano-biofilms” of *Candida albicans* were printed in a single study utilizing microarray printing techniques to create 1200 tiny droplets of 30 nL each. When compared to normally grown Candida albicans, the “nano-biofilms” formed by *Candida albicans* were determined to be genetically and morphologically identical. Subsequently, 28 combinatorial synergistic antifungals were found to be effective against the microdroplets when tested for drug sensitivity.

The methodology involves testing the activity of 28 combinatorial synergistic antifungal agents against fungal cells suspended in microdroplets. The first step is the preparation of a suspension, either by microfluidic techniques or microarray technology, which contains suspended fungal cells in an appropriate growth medium. Then, selected antifungal compounds will be prepared since these compounds would likely exhibit some form of synergy. Directly, the drug is added to the droplets while the antifungal drugs diffuse into the droplet environment from the agar plates in which it was stored. Microdroplets were left in growth conditions for fungi while ensuring the cells were within the critical incubation period for their interaction with antifungal agents. After incubation, the activity of each agent is determined either by colony-forming unit counts or biochemical assays assessing fungal growth. The outcomes are then evaluated to determine which pairs of antifungal agents exhibit synergy, which means a growth reduction greater than that resulting from individual agents. These promising combinations would then be further experimented upon to prove their potential efficacy and consistency, further leading to the advancement of handling fungal infection treatments, which are currently resistant to present antifungal drugs.

In addition, microarrays are a useful tool for speeding up the discovery of novel metabolic pathways and developing novel drugs against well-established antimicrobial drug-resistant organisms (ADROs). Liquid droplets are easily damaged in studies, and secondly, they can only be interrogated in a static position on a Petri plate. Gel droplets protect cells from damage and provide a stable experimental environment, but the optimal conditions for investigating flow remain to be determined for microarray technology [99].

## 12. Micromachined Devices

The grid layouts used in microfabrication allow for predictability and the use of readily available automated software for scoring growth, both of which contribute to the technology’s high level of accuracy [95]. There is a wide range in mechanical strength, permeability, biocompatibility, and surface charge among the materials utilized to make these gadgets [175].

An unanticipated advantage of these microdevices is their portability, allowing in situ incubation in locations other than a laboratory. Hence, rather than creating and managing artificially altered growth circumstances, we may rely on the inherent microenvironment to facilitate the cultivation of previously unattainable species [176]. Kaeberlein et al. [177] proposed in the early 2000s that it would be possible to isolate and culture microorganisms without understanding the precise components necessary to sustain the establishment of a given ecosystem by just employing their natural milieu. Since this was the case, they created diffusion chambers by placing an agar matrix containing an ambient sample between two membranes with pores of 0.03 m [178].

The ichip is induced by dilution-to-extinction of the ambient sample, resulting in the development of monocultures without the need for manual picking and isolation [179]. Many phylogenetically unique species, including those discovered in human mouths [180] and saltwater [181], have never been cultured using conventional methods. However, since the ichip was introduced, they have been cultivated and isolated.

**Figure 4:**
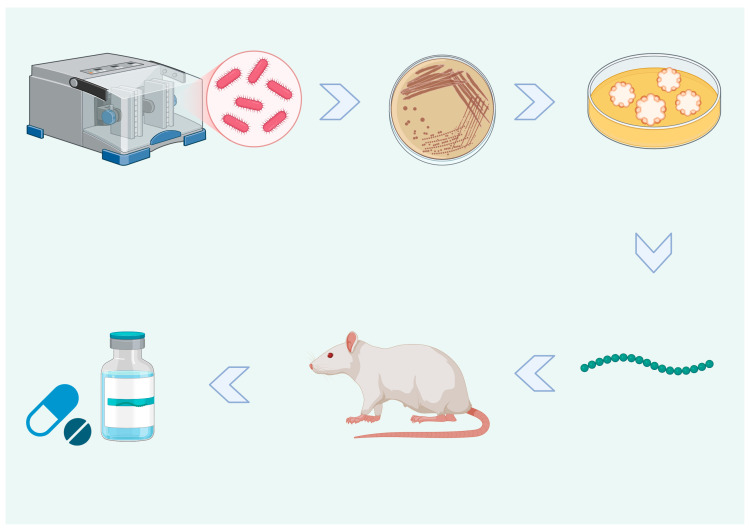
Teixobactin was identified during the isolation and passage of Eleftheria terrae, a novel proteobacteria.

Recently, novel antibiotics such as Novo10 and Neocitreamicin I and II have been identified thanks to the successful cultivation of rare environmental species utilizing the specified diffusion chips [182,183]. As shown in Figure 4, Teixobactin was identified during the isolation and passage of Eleftheria terrae, a novel proteobacteria [184]. It effectively killed Gram-positive bacteria, even those that had developed resistance to antibiotics.

Cultures of previously unknown species were established in a soil sample from the environment using the ichip. Antibiotic activity against S. aureus was tested by culturing various species onto agar plates. The in vitro efficacy of bioactive compounds against a wide range of pathogens was determined after their extraction, purification, and characterization. Using this method, researchers found teixobactin, a new antibiotic molecule that prevented any mutants from becoming resistant in early testing. In contrast, teixobactin showed promising results against resistance in initial tests; more studies are required to confirm long-term efficacy [184].

## 13. Single Emulsion Droplet Microfluidics

Droplet microfluidics is a rapidly advancing technology that has emerged increasingly into antimicrobial testing; these methods offer precise control of the experimental conditions and enable the analysis of microbial responses towards many different antimicrobial agents with high throughput. These microfluidic devices can generate tiny and discrete droplets, each functioning as an individual reaction chamber capable of encapsulating cells, reagents, or nutrients. Among the greatest advantages that can be reaped from the application of droplet microfluidics is the ability to produce microenvironments with more nearly natural conditions. It can provide the closest assessment of how microbes react and behave towards antibiotics. Droplets may have perfect control over the droplet size and the constituents that could be varied while examining the dose-response curves under highly controlled conditions. This feature helps establish MICs and, by implication, can study the synergy between a group of drugs. Thus, it provides a potent technique for discovering effective agents against a particular pathogen. Applications in droplet microfluidics have been used in testing the efficacy of new antibiotics for a broad spectrum of bacteria, even those not readily cultured or resistant to previous treatments. The technology is also useful for the fast screening of antimicrobial peptides or natural products, assisting in identifying new antimicrobial candidates. Scientists can learn how bacteria respond to antibiotics and the development of resistance or the activation of stress responses by observing microbial interactions and mechanisms of resistance in droplets in real time.

Because each droplet functions as a miniature bioreactor, droplet microfluidics has recently gained importance for studying cells. The culture medium often has length scales similar to prokaryotic and eukaryotic cells. Therefore, droplets ranging in size from pico- to microliters facilitate the fast passage of gases, nutrients, metabolic waste, and similar substances [97].

Beneyton et al. [185] employed a microfluidic system to conduct a screening process for the enzymatic production of amylase by the filamentous fungus *Aspergillus niger*. The droplets were generated at a frequency of 80–90 droplets per second and had a volume in the nanoliter range (10–18 nL). Agar droplets have also been widely used for cell confinement, in addition to the liquid, aqueous phase often associated with single emulsions. During encapsulation, the agar syringe is kept liquid by submerging it in a water bath that is heated to a regulated temperature [186]. Alkayyali et al. [187] created the Microbe Domestication Pod (MD Pod) to cultivate marine samples separated within agarose beads in a single emulsion. After the gel beads are injected inside the Pod, the device may be placed back in its natural home to continue its development.

Silicon molds can be etched, embossed, or lithographed using various methods available due to microelectromechanical system (MEMS) microfabrication processes. Soft photolithography, pioneered by Xia and Whitesides, is currently one of the most popular fabrication processes. In soft lithography, a master mold of the necessary microfluidic device is made of photo-cross-linkable polymers rather than silicon wafers [188]. Commercially available chips may be better for exploratory research, especially with environmental materials. Several university-based public foundries have been established to facilitate customization and ease of use in the fabrication process by enabling users to submit their designs for microfabricated chips without needing specialized training or financial outlay [189].

## 14. Double Emulsion Droplet Microfluidics and Polymer-Based Nano Cultures

Microfluidic channels with hydrodynamic pressure flow and co-flow geometry can create double-emulsion droplets, which function similarly to single-emulsion droplets. The cell inoculum is in an aqueous phase at the system’s center. The hydrophobic, hydrophilic intermediate phase produces double emulsion droplets, which are stabilized in solution by a surfactant in a continuous watery (hydrophilic) phase.

Because double-emulsion droplets partition chemical reactions into nanoliter bioreactors, droplet microfluidics enables high-throughput research. Products that have passed the chemical test can be separated using fluorescence-activated cell sorting. Zinchenko et al. [190] demonstrated the platform’s efficacy by identifying enhanced cellular clones capable of producing catalytically active enzymes from 106 inactive enzymes in 10 m droplets. Screening many separate assays with high signal quality is possible by compartmentalizing them in water-in-oil emulsion droplets. Droplet production and sorting using microfluidic devices is possible; however, these technologies are unsuitable for use in environments where operators lack the necessary expertise. This section lays out the processes required to create monodisperse double-emulsion droplets in two stages using microfluidic devices that have surfaces with hydrophobic and hydrophilic properties, respectively. Commercial flow cytometers are well-suited for quantitative examination and sorting of the produced double-emulsion droplets. Experiments showing the method’s efficacy in enrichment ultimately lead to successfully isolating catalytically active clones from a pool of 100,000 times more variations with poor activity. Additional steps can be integrated into the modular workflow, such as heat inactivation to stop the encapsulated lysate assay reactions, droplet size contraction to concentrate the contents, and storage at −80 °C for discontinuous workflows. This allows for ready control of selection stringency. The ability to regulate screening settings in this way will enable protein engineers to easily harness the power of protein libraries housed in droplets following an easy-to-follow approach.

Modifying the polymeric membrane to fit the specific structural requirements of different uses is possible. PDMS’s high mechanical toughness and elasticity make it an important material; its Young modulus is around 0.5 to 3 MPa [191]. While this aids in producing robust capsules for cell encapsulation, it complicates subsequent processing steps, such as emptying the capsules.

## 15. Applications of Microbial-Based Microsystems and Perceived Challenges

Optical transparency allows for the study of phylogenetic responses in isolated colonies that have been stimulated chemically. Observing increasingly complex consortia to examine intra- and inter-species interactions is vital in differentiating symbiotic and antagonistic behaviors across species and pathogenic switching in opportunistic illnesses.

Many difficult-to-manage species, however, have unidentified secondary metabolite needs [192]. Microorganisms can be cultivated in engineered microsystems, even in extreme environments that would have previously been impossible. *Clostridium difficile* was deemed an “Urgent Threat” [193], according to the 2019 report on Antimicrobial Resistance Threats from the United States Centers for Disease Control and Prevention (CDC). Monocultures provide a new perspective for studying the intricate interactions between gut microbiota and C. difficile in a low-oxygen environment by allowing optical access under a microscope.

The purpose of these ecosystems is not confined to the production of so-called “unculturable” species. Many applications that previously were impossible to accomplish with the level of control, accuracy, and resolution required to identify study targets have been made possible thanks to the development of miniaturizing culturing techniques [194]. Low-throughput, expensive approaches are often connected with drug and enzymatic metabolite screening in the biotechnology industry [195]. Microfluidics is superior because it streamlines the process by shrinking everything to a microscopic size. Rapid turnaround time (a few hours instead of days) reduces the overall expense of screening procedures. Diagnostic tools, biological assays, and analytical tools in the pharmaceutical business can greatly benefit from the replicability and automation provided by microfluidics [196].

Materials design is an example of a more specialized difficulty encountered during gadget production. According to Aleklett et al. [97], unlike naturally occurring habitats, PDMS, an essentially hydrophobic elastomer, does not offer cells a heterogeneous setting where they can be mechanically manipulated. However, the hydrophilic surface modifications induced by oxygen plasma treatment of PDMS are only transitory, as the surface quickly reverts to its lower free energy state due to the migration of oligomers inside the PDMS network [197].

## 16. Advantages and Disadvantages of Antibiotics Derived from Bacterial Sources as Compared to Fungal Sources

Antibiotics derived from bacterial sources, such as *Streptomyces* and other actinobacteria, and those from fungal sources, like *Penicillium*, both play vital roles in combating microbial infections, but they have distinct advantages and disadvantages. Bacterial-derived antibiotics, such as streptomycin and tetracycline, often exhibit a broader spectrum of activity, making them effective against a wide range of bacterial pathogens. Additionally, many bacterial sources produce complex bioactive molecules with unique mechanisms of action, offering potential solutions to multidrug-resistant infections. However, one disadvantage is that bacteria can quickly evolve resistance to these antibiotics due to horizontal gene transfer, leading to reduced efficacy over time.

On the other hand, fungal-derived antibiotics, such as penicillin and cephalosporins, have a proven track record in clinical medicine and tend to have fewer side effects for patients than some bacterial antibiotics. Fungal antibiotics, particularly beta-lactams, are highly effective in inhibiting cell wall synthesis in bacteria, a mechanism less prone to resistance development. Nevertheless, fungal-derived antibiotics often have a narrower spectrum of activity, limiting their use to specific bacterial infections. Additionally, fungi are less prolific producers of novel antibiotic classes than bacteria, which could hinder the future discovery of new drugs from fungal sources. In summary, while both bacterial and fungal antibiotics are crucial in medicine, bacterial sources offer diversity and a broader spectrum. In contrast, fungal antibiotics provide established, highly effective treatments, albeit with limited scope.

## 17. Conclusions

In the current era of antimicrobial resistance, the diversity of chemicals found in bacteria and other microorganisms and their powerful action is important. Expanding our search to include these organisms will lead us to potentially game-changing antimicrobials with mechanisms of action not yet seen in existing drugs. That way, antimicrobial resistance can be drastically decreased. To fully understand the influence of microbial diversity and activity on various levels of interactions and system fluxes, it is necessary to perform experiments across several scales, ranging from individual organisms to communities and entire ecosystems. To accurately portray the Earth as a whole, models of the biosphere must account for both the biotic (consisting of bacteria, plants, and organic matter substrates) and abiotic (including mineral surfaces, ocean physics, and chemistry) environments in which microbes operate. There are now fascinating and unique ways to explore “microbial dark matter” and the accompanying microbial dynamics because of the downsizing of culturing technologies. Precise instruments created by microfabrication techniques are used to investigate the microenvironment of cells. Instead of using conventional nutrient-rich liquids, these devices utilize natural surroundings that exist everywhere.

## Figures and Tables

**Table 2:** Bacterial origins of chemicals that inhibit fungal growth.

Microorganism	Susceptible Organism(s)	Compound(s)	Reference
*A. hibiscus*	*Aspergillus* spp., *Candida* spp.	Pradimicins A, B, C	[56]
*Actinoplanes* spp.	*T. mentagrophytes*	Purpuromycin	[57]
*B. cereus*	*Aspergillus* spp., *Saccharomyces* spp., and *C. albicans*	Azoxybacilin, Bacereutin, Cispentacin, and Mycocerein	[58]
*M. neiheumicin*	*S. cerevisiae*	Neihumicin	[59]
*Micromonospora species*	*A. cladosporium*, *C. albicans*, and *Cryptococcus* spp.	Spartanamycin B	[59]
*Micromonospora species SCC 1792*	*Dermatophytes* and *Candida* spp.	Sch 37137	[60]
*Micromonospora species SF-1917*	*R. solania*	Dapiramicins A and B	[61]
*B. subtilis*	*Phytopathogens*	Iturin A	[62]
*B. lichenformis*	*Mucor* spp., *Microsporum canis*, *Sporothrix* spp.	Fungicin M-4	[63,64]

**Table 3:** Biochemical substances derived from fungi that exhibit antimicrobial action.

Microorganism	Antimicrobial Activity	Compounds	Reference(s)
*Hormonema* spp.	Aspergillus spp., Candida spp.	Enfumafungin	[93]
*F. calocera*	*Mucor plumbeus*, *Candida tenuis*	Favolon	(Chepkirui et al., 2016) [94]
*C. comatus*	*P. aeruginosa*	Coprinuslactone	(Hyde et al., 2019) [95]
*Sanghuangporus* spp.	*C. albicans*, *S. aureus*	Microporenic acid A	(Chepkirui et al., 2018) [96]
*Aspergillus terreus*	Influenza A virus	Rubrolide S	(Zhu et al., 2014) [97]
*Cladosporium sphaerospermum*	Influenza A H1N1	Cladosin C	(Wu et al., 2014) [98]
*Penicillium* sp.	HCV, HIV	β-hydroxyergosta-8,14,24 (28)-trien-7-one, Trypilepyrazinol	(Li et al., 2019) [99]

## Data Availability

All information produced or examined in this research is incorporated within this published article.
